# Porous Carbon Microparticles as Vehicles for the Intracellular Delivery of Molecules

**DOI:** 10.3389/fchem.2020.576175

**Published:** 2020-10-14

**Authors:** Luis M. Magno, David T. Hinds, Paul Duffy, Rahul. B. Yadav, Andrew D. Ward, Stan W. Botchway, Paula E. Colavita, Susan J. Quinn

**Affiliations:** ^1^School of Chemistry, University College Dublin, Dublin, Ireland; ^2^School of Chemistry, Trinity College Dublin, Dublin, Ireland; ^3^Rutherford Appleton Laboratory, Central Laser Facility, Science & Technology Facilities Council, Didcot, United Kingdom

**Keywords:** porous carbon, microparticles, cells, delivery, fluorescent

## Abstract

In this study the application of porous carbon microparticles for the transport of a sparingly soluble material into cells is demonstrated. Carbon offers an intrinsically sustainable platform material that can meet the multiple and complex requirements imposed by applications in biology and medicine. Porous carbon microparticles are attractive as they are easy to handle and manipulate and combine the chemical versatility and biocompatibility of carbon with a high surface area due to their highly porous structure. The uptake of fluorescently labeled microparticles by cancer (HeLa) and normal human embryonic Kidney (HEK 293) cells was monitored by confocal fluorescence microscopy. In this way the influence of particle size, surface functionalization and the presence of transfection agent on cellular uptake were studied. In the presence of transfection agent both large (690 nm) and small microparticles (250 nm) were readily internalized by both cell lines. However, in absence of the transfection agent the uptake was influenced by particle size and surface PEGylation with the smaller nanoparticle size being delivered. The ability of microparticles to deliver a fluorescein dye model cargo was also demonstrated in normal (HEK 293) cell line. Taken together, these results indicate the potential use of these materials as candidates for biological applications.

## Introduction

The ability to engineer robust particles tailored for diagnostic and therapeutic applications is an active area of research and requires the presence of multifunctional surfaces able to direct, detect, deliver and signal processes (Ma et al., 2011). For this purpose porous particles are attractive candidates due to their high adsorption capacity (Ariga et al., 2007; Tian et al., 2007; Lu et al., 2010). These particles have found use in a wide range of applications including controlled drug release (Kim et al., 2008; Slowing et al., 2008; Zhao et al., 2009; Fang et al., 2010), cellular delivery (Yan et al., 2006; Nakayama-Ratchford et al., 2007; Liu, J. et al., 2009; Gu et al., 2011), energy storage (Guo et al., 2009; Liu, H.-J. et al., 2009; Adcock et al., 2013), catalysis (Chen et al., 2012; Figueiredo, 2013), water treatment (Chen et al., 2012; Li et al., 2016), and solar cells (Chen et al., 2009). To date, silica has emerged as the mesoporous material of choice for cell delivery applications (Lu et al., 2007; Argyo et al., 2013; Zhang J. et al., 2014). However, the chemical versatility and biocompatibility, has led to substantial research in the areas of biotechnology and biomedicine where it is already used as a coating in biodevices and implants (Roy and Lee, 2007).

Carbon nanomaterials stand out as an extremely versatile family of nanomaterials with a range of highly desirable properties for applications in biology. Carbon surfaces often display excellent performance as blood- and tissue-contacting materials due to a combination of frictional and chemical stability properties that can be tailored to meet diverse requirements: (Vasconcelos et al., 2020) for instance, thanks to their properties and safety profile, graphitic carbon materials are currently used as heart valve coatings while diamond-like carbons find applications in joint replacements (Gott et al., 2003; Grill, 2003; Lemons). Carbon surfaces are highly resistant to the corrosive environment of physiological fluids and, contrary to most metal and alloy surfaces, they are not prone to leaching and pitting/dissolution, which might result in cytotoxicity and adverse health effects (Roy and Lee, 2007). In addition to desirable surface chemistry, the morphology and nanoarchitecture of carbon materials can be adapted to achieve diverse functionality; a wide range of nanoallotropes can be synthesized from low-cost and earth-abundant organic precursors, that can be used to vary both morphology and surface chemistry of the resulting nanomaterial (Georgakilas et al., 2015; Domínguez et al., 2020). Therefore, carbon offers an intrinsically sustainable platform material for tailoring and modulating morphology, bulk and surface properties in order to meet the multiple, and complex requirements imposed by applications in biology and medicine.

Carbon nanomaterials have been extensively studied with notable examples in the literature of outstanding performance in sensing (Baptista et al., 2015) and imaging applications (Bartelmess et al., 2015). Suitably modified single-walled carbon nanotubes (SWNTs) have been functionalized for cell delivery (Liu et al., 2007), though there are many challenges associated with their purification and manipulation (Biju, 2014). Highly sorbent activated carbon has been used as an amorphous drug carrier for paracetamol and ibuprofen (Miriyala et al., 2017). In particular, the low-toxicity of spherical nanocarbon materials is of increasing interest for cellular imaging and drug delivery applications (Wang et al., 2014). We have recently shown spherical carbon nanohorns to be effective for cell imaging and cell delivery (Devereux et al., 2018, 2019) and the related sp^2^ carbon nano-onions have also been studied (Bartkowski and Giordani, 2020). Porous spherical carbon microparticles (CμP) are easy to handle and purify, and their synthesis from inexpensive precursors is scalable and simple (Skrabalak and Suslick, 2006; Fang et al., 2010). Particles of variable size can be readily prepared ranging from 200 nm to 1 μm diameter with tunable surface area in the region 500–1,000 m^2^ g^−1^. The high specific surface makes the materials ideal supports for catalysis (Metz et al., 2015; Dominguez et al., 2018), while the rich functional chemistry of carbon surfaces allows the use of standard routes including bioconjugate techniques to engineer the chemical nature of the surface (Sun et al., 2006; Duffy et al., 2012). These features facilitate the physical entrapment of large quantities of cargo, the covalent conjugation of small molecules or biological species and also the capability of directing delivery through surface modification. These facts combine to make porous carbon particles and microparticles (CμPs) highly attractive agents for intracellular delivery (Kim et al., 2008).

**Scheme 1:**
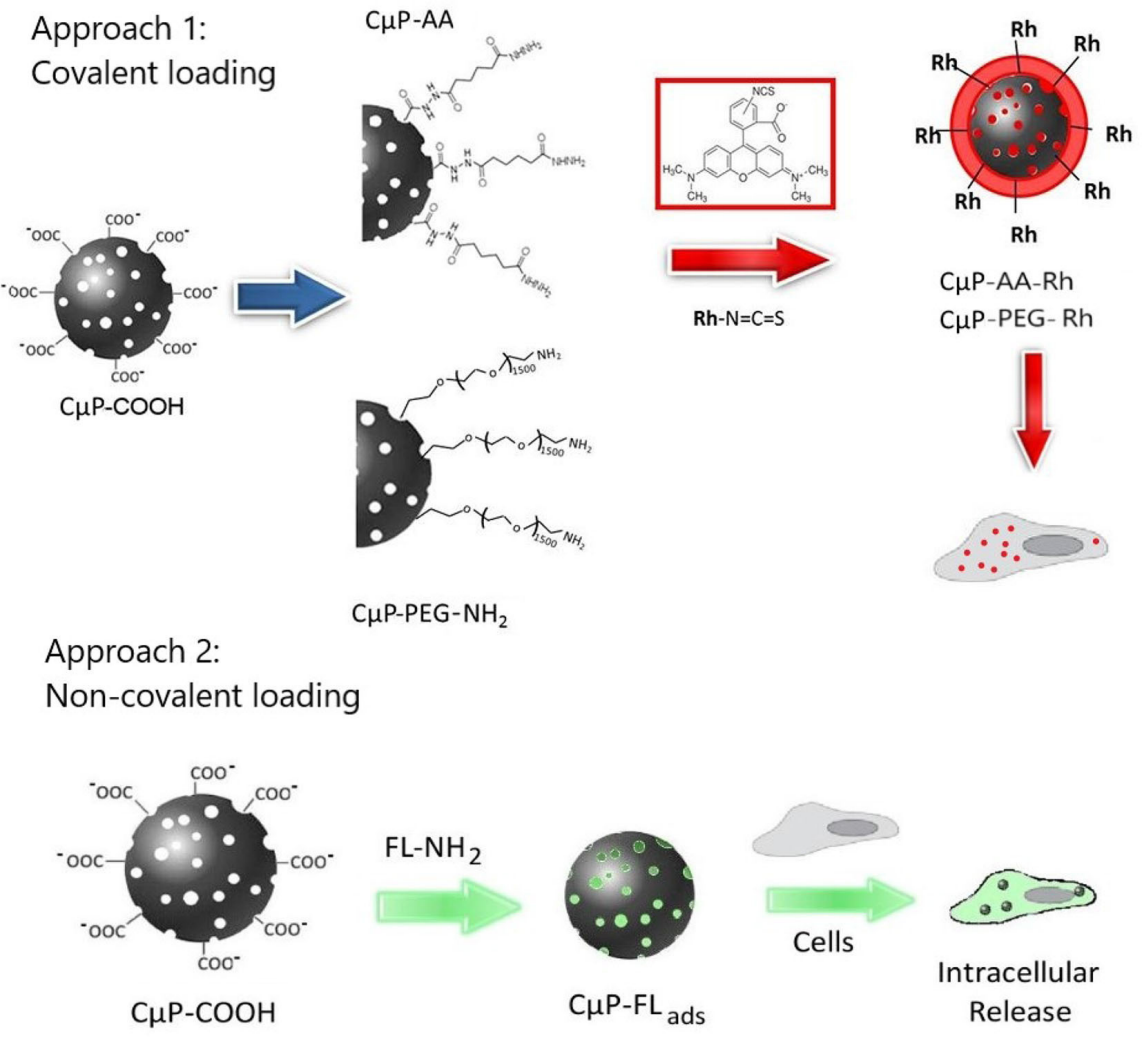
Schematic diagram showing the cellular interactions of CμPs modified with fluorescent dye molecules through approach 1, the covalent and approach 2, non-covalent surface functionalization.

In our previous study we demonstrated that dye-labeled 700 nm porous CμPs, functionalized by amide coupling of 6-aminofluorescein directly to the –COOH surface groups, could be internalized by cancer cells in the presence of a transfection agent (Duffy et al., 2012). Confocal fluorescence images suggested preferential clustering of the particles in specific subcellular structures. In this study we now compare the cellular uptake behavior of similar sized “large” 690 nm (L-CμPs) and smaller particles 240 nm (S-CμPs). In this work the inherently non-luminescent CμPs are covalently functionalized with rhodamine B fluorescent dye via an adipic linker molecule to allow imaging by confocal microscopy (Scheme 1). The use of this spacer molecule allows separation of the dye from the particle surface. In addition, the role of surface functionalization is also examined by investigating the effect of PEGylation on the cellular uptake, which is known to aid the dispersibility of particles in water, improve the efficacy and reduce immunogenicity (Jokerst et al., 2011; Kolate et al., 2014; Suk et al., 2016). To investigate the use of these particles for cellular delivery, we also report on the ability of CμPs to deliver a model payload. Negatively charged CμP-COOH particles were non-covalently loaded with a highly fluorescent amino-fluorescein dye to prepare CμP-FL_ads_. The ability of these particles to deliver their model cargo to cells was then investigated (Approach 2 in Scheme 1). This preliminary study shows that cells tolerate a range of sizes of porous carbon particles, which have potential as delivery agents for therapeutics.

## Experimental Section

### Chemicals and Materials

All chemicals unless stated otherwise were used as received without further purification, nitric acid (64–66%, Sigma), sodium chloride (Fisher), sodium carbonate (VWR). “SnakeSkin” dialysis tubing (regenerated cellulose, 3,500 MWCO), was supplied by Thermo scientific. All water used for chemical modifications was deionized (diH_2_O); sterile water was used for solutions used in cell incubation.

### Synthesis

#### Synthesis of CμPs

Carbon microspheres with two different sizes were synthesized via ultraspray pyrolysis (USP) following reported methods (Skrabalak and Suslick, 2006; Duffy et al., 2012). Briefly, a 1.7 MHz piezoelectric disk placed at the bottom of a flask was used to generate a mist from an aqueous solution of lithium dichloroacetate. The smaller (S-CμP) particles were generated from a 0.125 M solution of lithium dichloroacetate and the larger (L-CμP) particles were generated from a 1.00 M solution of lithium dichloroacetate. The mist was carried by a flow of Ar into a tube furnace, where the organic salt was pyrolyzed at 700°C. Particles were collected in a bubbler containing deionized water, filtered and washed with copious amounts of water prior to further characterization.

#### Synthesis of CμP-COOH

CμPs were suspended in water and refluxed in 5 M nitric acid at 80°C for 2 h. Following the reaction, the particles were washed with water and purified by dialysis against water (1% NaCl).

#### Synthesis of CμP-AA

S and L-CμP-COOH (2.5 mg) were dispersed in an aqueous solution of adipic acid dihydrazide (16.0 mg) in 0.1 M sodium phosphate (pH 7.2) at RT under stirring. To this dispersion 8.0 mg of N-Ethyl-N'-[3-dimethylaminopropyl] carbodiimide (EDC) was added and the reaction was stirred at room temperature for 4 h (Hermanson, 2008). The microparticles were washed with water by repeated centrifugation and purified by repeated dialysis against water (1% NaCl) for 3 days.

#### Synthesis of CμP-PEG-NH_2_

Prior to reaction, CμP-COOHs were soaked in 50 mM 2-(N-morpholino)ethanesulfonic acid (MES) buffer (pH 6) for 24 h. Particles were then concentrated and re-suspended in 0.5 mL of MES buffer containing 10.0 mg of EDC and 10.0 mg of N-hydroxysuccinimide (NHS) and vigorously agitated for 15 min. Subsequent to this, the reaction mixture was washed twice by centrifugation, at 13,300 rpm, with buffer and re-suspended in 0.5 mL MES buffer containing 5.0 mg of O,O'-Bis(3-aminopropyl)polyethylene glycol 1,500. The reaction mixture was agitated for 4 h at RT. The particles were then washed with diH_2_O, re-suspended in diH_2_O and purified by dialysis against water (1% NaCl) for 3 days.

#### Synthesis of CμP-AA-Rh and CμP-PEG-Rh

The amine modified particles (CμP–AA/CμP-PEG-NH_2_) were soaked for 24 h in an aqueous solution of 0.1 M sodium carbonate (pH 9.0). In a darkened lab 50 μL of tetramethylrhodamine-5-isothiocynate (1 mg mL^−1^) in DMSO was added to 200 μL of the amino modified carbon particle dispersion (1 mg mL^−1^) in 0.1 M sodium carbonate (pH 9.0). The reaction was allowed to proceed for 8 h at 4°C in the dark. The final product was washed repeatedly with water and purified by dialysis against water (1% NaCl). The same protocol was used with fluorescein-5-isothiocynate to prepare CμP-AA-Fl and CμP-PEG-Fl.

#### Synthesis of CμP-FL_ads_

Fluorescein loaded microparticles were prepared by adding 300 μL of 6-aminofluorescein (1 mg mL^−1^) in DMSO to a dispersion of CμP-COOH at pH 7.0 and incubating at RT overnight. The non-adsorbed 6-aminofluorescein was removed by repeated centrifugation and washing with water.

### Particle Characterization

Scanning Electron Microscopy (SEM) was carried out on Zeiss Ultra Plus microscope using the In-lens detector. The hydrodynamic radius and ζ-potential of the particles were measured using a Malvern Nano HT Zetasizer. The particles (≥10 μg mL^−1^) were suspended in a 10 mM aqueous solution of NaCl at pH 7.0 for at least 12 h prior to measurement. A refractive index of 1.8 was used for DLS size modeling. The electrophoretic mobility of the sample was measured and converted into ζ-potential by applying the Henry equation and Smoluchowski approximation (Dispersion Technology Software 4.20 Malvern). Fluorescence (photoluminescence) spectra were measured on a Varian, Cary Eclipse fluorescence spectrophotometer.

### CμP-FL_ads_ Adsorption Study

The loading/adsorption of aminofluorescein by carboxylated microparticles was monitored over a 24 h period. One hundred microliter of 6 aminofluorescein (1 × 10^−6^ M) was added to 2.5 mL of an aqueous dispersion (pH 7) of L-CμP-COOH. The RT emission (λ_exc_ = 495 nm) was measured over 24 h at 30 s intervals. The measurement was then repeated in the absence of microparticles.

### Cell Culture

Human embryonic kidney (HEK293) and human epithelial carcinoma (HeLa) cells were purchased from Prochem (EACCC, UK) and cultured under the following conditions: cells were cultured in Eagle's minimal essential media (EMEM) supplemented with 10% fetal calf serum–(American, Fetal), 2 mM L-glutamine, 100 units mL^−1^ penicillin G sodium and 100 μg mL^−1^ streptomycin. The cells were grown by incubating at 37°C with 5% CO_2_ in humidified air until the culture reached 70% confluency before replating at 2 × 10^5^ cells per dish, in 35 mm glass bottom dishes (MatTek Corporation, USA) containing 2–3 mL of medium prior to confocal imaging. Fourteen hours later membrane dye labeled cells were prepared by culturing in the presence of 200 μL of 1 μM 1,1'-Dioctadecyl-3,3,3',3'-tetramethylindocarbo-cyanine iodide (DiI, Invitrogen) in serum-free EMEM media for 8 min at 37°C with 5% CO_2_. Aqueous microsphere suspensions (0.04 mg mL^−1^) were prepared using sterile water. The particles (50 μL) were then added to 200 μL OptiMem I reduced serum media and when referred 12 μL of FuGENE®, a cationic lipid transfection agent, was added directly into the solution and mixed. After incubation for 20 min this mixture was added to cells and incubated overnight at 37°C. Cells were washed three times with phosphate buffered saline solution (PBS) prior to imaging.

### Confocal Laser Scanning Microscopy

High-resolution confocal images were obtained using a Nikon confocal laser scanning microscope, EC1-Si (CLSM) attached to an inverted Nikon TE2000-U microscope and a 60 × water immersion objective. An argon ion and a helium-neon laser operating at 408, 488 and 543 nm, respectively, were used alternately with line switching using the multi-track facility of the CLSM. Images were collected using a 488/543 dichroic beam splitter and a 512–530 band pass filter to detect fluorescence from fluorescein labeled particles, using a 560–615 band pass filter to detect fluorescence from rhodamine modified particles and using optical white light transmission.

## Results and Discussion

### Preparation and Characterization of Carbon Microparticle Systems

**Figure 1:**
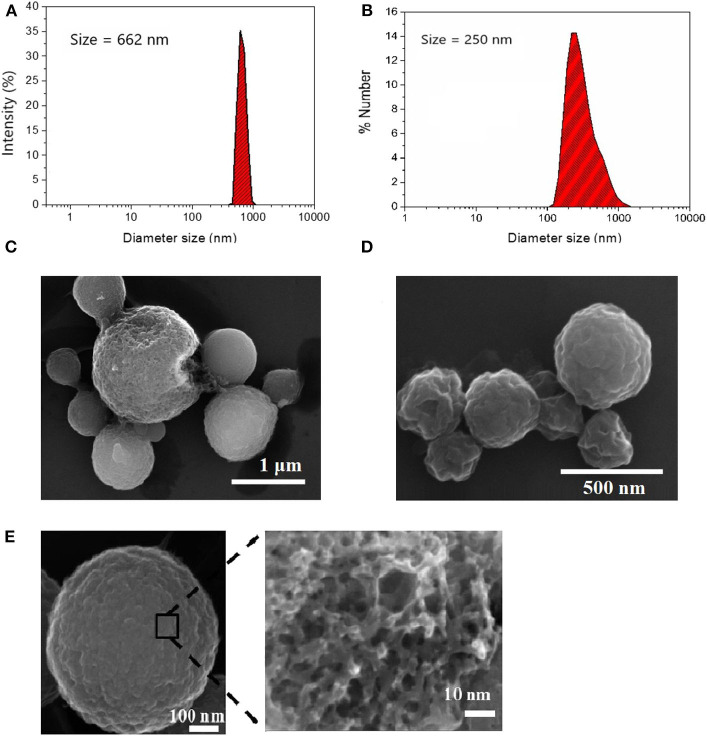
Size distribution of **(A)** L-CμP and **(B)** S-CμP particles as determined by Dynamic Light Scattering (DLS). Samples were recorded in distilled deionized water (ddiH_2_O) at 25°C; and Scanning Electron Microscopy (SEM) images of **(C)** L-CμP and **(D)** S-CμP synthesized via ultraspray pyrolysis. **(E)** SEM of L-CμP-COOH prepared by refluxing L-CμP in 5 M HNO_3_ for 2 h at 80°C.

Spherical porous carbon microparticles were prepared via ultraspray pyrolysis (USP) of a lithium dichloroacetate precursor at two different sizes following our previous work (Duffy et al., 2012) and the method of Skrabalak and Suslick (2006) Figures 1A,B show particle size distributions obtained via DLS using 1.00 M and 0.125 M precursor solutions, respectively. The mean particle diameter of the particle synthesis using size distribution was determined in solution via DLS yielding a population of 662 nm for the particles prepared at the higher concentration (1.00 M) and 250 nm for lower concentration (0.125 M) of precursor; this is in good agreement with the size determined from SEM analysis (Figures 1C,D). Surface oxidation of the CμPs was performed by treatment in 5 M nitric acid at 80°C for 2 h followed by repeated washing with water and purification by dialysis against saline solution (1% NaCl). SEM analysis revealed that oxidative treatment did not damage the particle morphology (Figure 1E).

The as purified L-CμP-COOH and S-CμP-COOH particles were found to be readily dispersed in water. The presence of negatively charged carboxylate groups on the purified sample was reflected in a decrease in ζ-potentials, which were measured at −30.0 ± 3.0 mV and −37.4 ± 2.2 at pH 7 for large and small particles, respectively (Table 1). The Raman spectra showed the presence of G and D bands characteristic of amorphous carbon materials. The G band (1,592 cm^−1^) arises from the in-plane vibration of sp^2^ carbon, while the D band (1,345 cm^−1^) results from a breathing mode of six-membered rings that becomes Raman active due to the presence of defects in the graphitic structure (Ferrari and Robertson, 2000). The I(D)/I(G) ratio was found to increase from 0.62 to 0.71 upon oxidation indicating an increase in defects, see Supplementary Figure 1 (Ferrari and Robertson, 2000; Duffy et al., 2012). Finally, the elemental composition of the oxidized particles was also examined using elemental analysis, which yielded carbon and oxygen contents of ca. 58% and 38%, respectively, with the balance attributed to hydrogen and nitrogen.

**Table 1:** Zeta potential measurements of surface functionalization ^[a]^.

**690 nm L-CμPs**	**ζ-Potential mV**	**240 nm S-CμPs**	**ζ-Potential mV**
L-CμP	−23.4 ± 1.0	S-CμP	−29.0 ± 2.0
L-CμP-COOH	−30.0 ± 3.0	S-CμP-COOH	−37.4 ± 2.2
L-CμP-AA	+15.8 ± 8.0	S-CμP-AA	−1.8 ± 0.6
L-CμP-AA-Rh	−11.0 ± 1.2	S-CμP-AA-Rh	−13.0 ± 1.4
L-CμP-PEG-NH_2_	−11.0 ± 1.2		
L-CμP-PEG-Rh	−24.0 ± 2.0		

*[a] All measurements in 10 mM NaCl aqueous solution, pH 7*.

**Figure 2:**
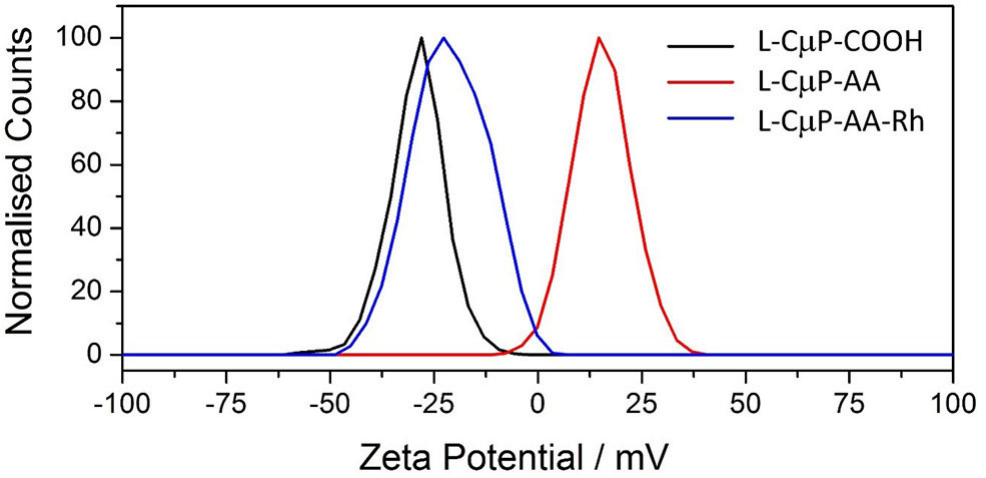
Zeta potential of L-CμP-AA, L-CμP-COOH and L-CμP-AA-Rh in 10 mM NaCl at pH 7.0.

The covalent and non-covalent loading of porous particles with fluorescent dye labels was carried out. Rhodamine B and fluorescein were chosen as they are standard dyes used for cellular imaging. The L-CμP-COOH particles were covalently modified with Rhodamine B through either a short adipic alkyl chain or a longer polyethylene glycol (PEG) linker (*n* = 1,500), as outlined in Scheme 1. In the first case, rhodamine B modified L- and S-CμPs were prepared by coupling adipic acid dihydrazide (AA) to the L/S-CμP-COOH surface to yield an amino terminated CμP-AA surface, which was then covalently coupled to tetramethylrhodamine-5-isothiocyanate using standard bioconjugate techniques to yield CμP-AA-Rh (Hermanson, 2008). The particles were then washed with water and purified by dialysis against saline (1% NaCl). This process was repeated until no emission from the rhodamine dye was detectable in the supernatant solution. The surface functionalization was monitored by ζ-potential measurements, which is an established method for monitoring nanoparticle modifications (Thielbeer et al., 2011). ζ-potential values were found to change after covalent functionalization with the adipic acid linker and the rhodamine B dye (Figure 2). This is consistent with a decrease in the density of negatively charged groups present at the particle surface after covalent functionalization. The dye functionalized particles were found to be readily dispersed in aqueous solution. Over time sedimentation of the particles was observed but was reversed with mild agitation.

In addition, the L-CμP-COOH were also modified using the PEG linker. The use of the longer linker did not result in the same magnitude of change in the zeta potential which suggested less effective functionalization (Table 1).

**Figure 3:**
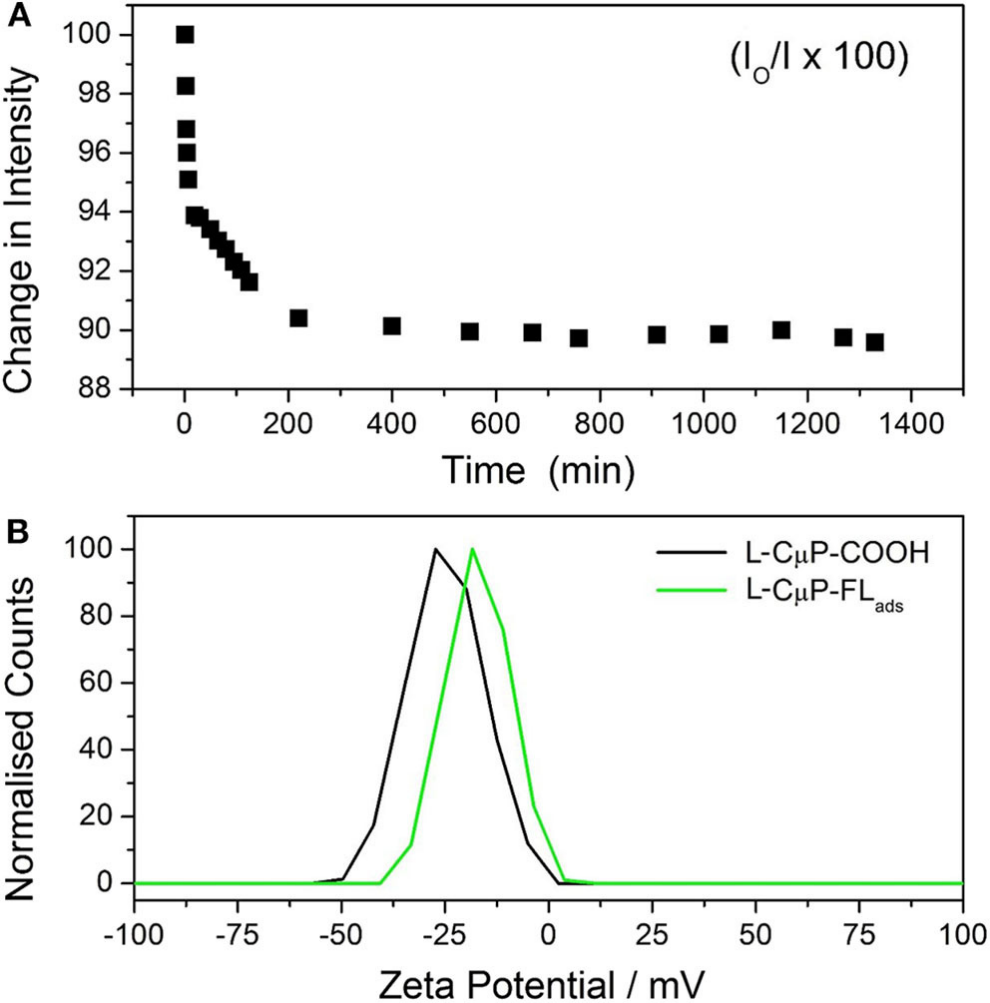
Adsorption of fluorescein dye at particles surface. **(A)** Time-dependent emission (520 nm) of 6-aminofluorescein upon incubation with L-CμP-COOH. **(B)** Zeta potential of L-CμP-COOH before and after adsorption of FL-NH_2_ at the surface to form and CμP-FL_ads_ in 10 mM NaCl at pH 7.0.

Carbon materials are highly sorbent and porous particles can adsorb large quantities of molecules. 6-aminofluorescein (FL-NH_2_) was chosen as the model cargo for cellular delivery, as its emission is readily imaged, it does not cause cell damage and has high affinity for the CμP-COOH surface due to a combination of its weak solubility in water and the presence of the amino group. The dye-loaded particles, L-CμP-FL_ads_ were prepared by incubating FL-NH_2_ with buffered aqueous L-CμP-COOH particles at pH 7, with mild stirring, for 24 h. This was followed by repeated centrifugation to wash the particles. The adsorption of the dye at the particle surface was followed by recording the emission of FL-NH_2_ at 520 nm (λ_exc_ = 495 nm) in the presence of L-CμP-COOH particles vs. time (Figure 3A). The loss of intensity with time is attributed to the adsorption of the dye at the L-CμP-COOH particle surface, with the majority of adsorption occurring in the first 30 min of incubation. No further loss in emission intensity was observed over the same period of time in the absence of particles. FL-NH_2_ adsorption at the particle surface is further supported by ζ-potential measurements which reveal a reduction in the surface negative charge for the L-CμP-FL_ads_ sample when compared with L-CμP-COOH (Figure 3B).

#### Cellular Uptake of the Larger Dye Labeled L-CμPs

**Figure 4:**
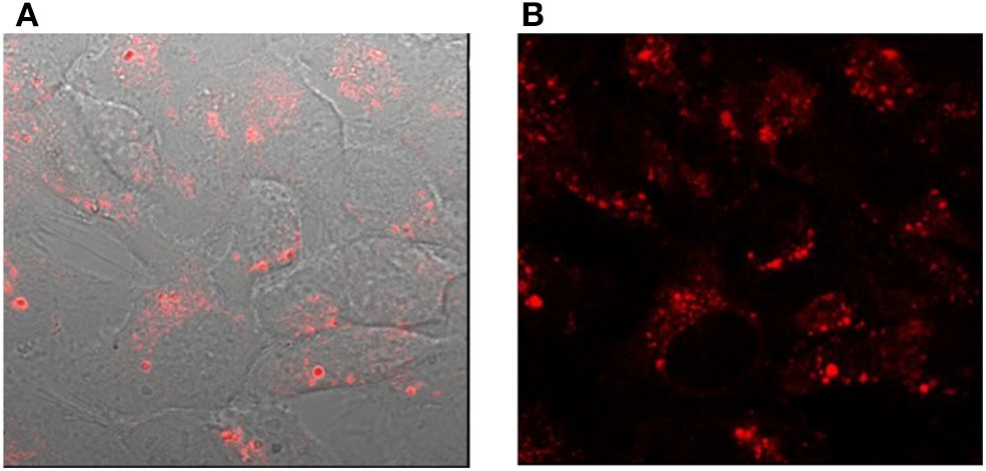
Confocal images of HeLa cells incubated with L-CμP-AA-Rh particles in the presence of the transfection agent **(A)** bright field image with fluorescence overlay and **(B)** fluorescence image.

The cellular studies were performed using a cancer cell line namely, human epithelial carcinoma (HeLa) cells and non-cancer human embryonic kidney cells HEK293. In particular, we were interested to investigate the cellular uptake in the presence and the absence of a transfection agent, which assists in the transport of materials to the cell. The transfection agent FuGENE® was used for this purpose, which is a non-liposomal agent with high efficiency of transporting macromolecules such as DNA, and have a low toxicity. The cellular internalization of L-CμP-AA-Rh and L-CμP-PEG-Rh particles was initially studied by pre-incubating the particles with the transfection agent FuGene. The dye-labeled particles were incubated with HeLa and HEK293 for 24 h. The cells were then imaged using confocal microscopy and using this technique the particle fluorescence was readily observed. For both particle sizes, confocal images revealed the majority of the particles to be internalized by the cells and to reside primarily in the cytoplasm and perinuclear region. This can be clearly seen in Figure 4 where bright field and fluorescence images are shown in the case of the L-CμP-AA-Rh particles incubated with HeLa cells. The appearance of strong localized fluorescence suggests that the particles are clustered preferentially in specific subcellular structures. This is in agreement with our previous results on the uptake of fluorescein labeled 700 nm CμPs performed in the presence of the same transfection agent (Duffy et al., 2012). The particle internalization was confirmed by Z-depth measurements and by overlaps of the bright field and fluorescence micrographs. Having demonstrated the ability to visualize the rhodamine labeled particles internalized using a transfection agent, we next considered the uptake without the transfection agent. In the absence of transfection agent neither HeLa nor HEK293 cell lines demonstrated the uptake of the larger particles. Instead the particles were observed to remain in the bulk solution or around the cell membrane (Supplementary Figure 2).

Non-specific interactions of particles with proteins and other biomolecules can lead to agglomeration of particles under cell-culture conditions and can also lead to binding to the cell membrane and the extracellular matrix (Cedervall et al., 2007). These processes lead to reducing the capacity of particles to act as delivery and imaging agents (Verma and Stellacci, 2010). This can be avoided by coating particles with molecules such as PEG, which is known to reduce non-specific binding of blood components such as proteins and macrophages (Pirollo and Chang, 2008). In this way the enhanced permeability and retention effect can be greatly improved and the blood circulation half-lives of PEGylated particles can be prolonged (Pirollo and Chang, 2008; He et al., 2010; Valencia et al., 2011). Furthermore, PEGylation is observed to reduce immunogenicity, shows good biocompatibility and aids in the dispersibility of particles in water (Jokerst et al., 2011; Kolate et al., 2014; Suk et al., 2016). PEGylation has been extensively used to enhance the delivery of fluorescently labeled nanoparticle (Ruan et al., 2014; Zhang, X. et al., 2014; Wang et al., 2015). For these reasons we next considered the uptake phenomena of PEGylated CμPs.

**Figure 5:**
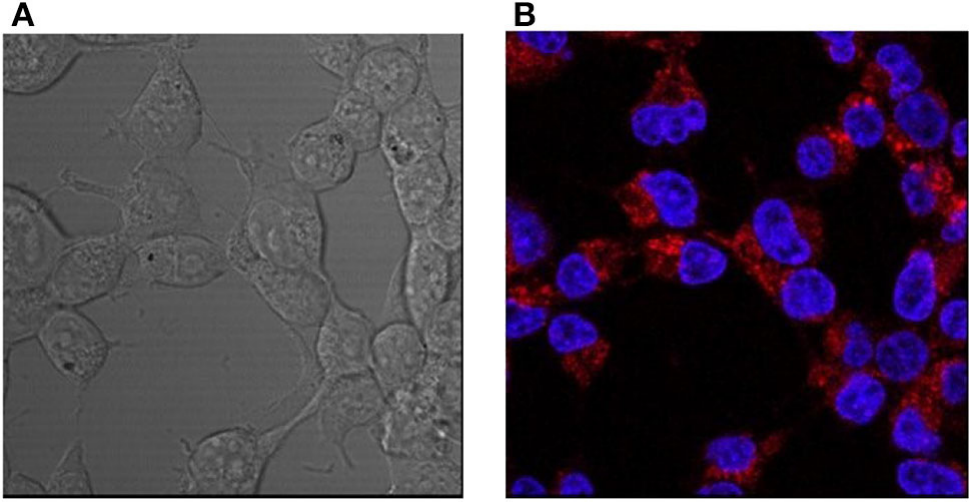
Confocal images of HEK293 cells incubated with L-CμP-PEG-Rh particles and co-stained with a DAPI dye. **(A)** Bright field and **(B)** fluorescence confocal images and the corresponding particles incubated without transfection agent.

HEK293 cells were incubated with L-CμP-PEG-Rh particles with and without transfection agent (FuGene). In the case of particles pre-incubated with FuGene the confocal fluorescence micrographs and Z-depth analysis shows that the particles entered into the cytoplasm of HEK293 cells (Supplementary Figure 3). Interestingly, cellular internalization of the L-CμP-PEG-Rh particles by HEK293 cells was also observed in the absence of the transfection agent (Figure 5). The volume analysis confirms that particles are localized in the cytoplasm (Supplementary Figure 3). While the number of particles internalized is lower in the absence of transfection agent. In contrast to adipic acid modified particles, PEG modified particles tend to stay in the cytoplasm and almost no particles were observed in the nuclear region.

#### Cellular Uptake of the Smaller Dye Labeled S-CμPs

**Figure 6:**
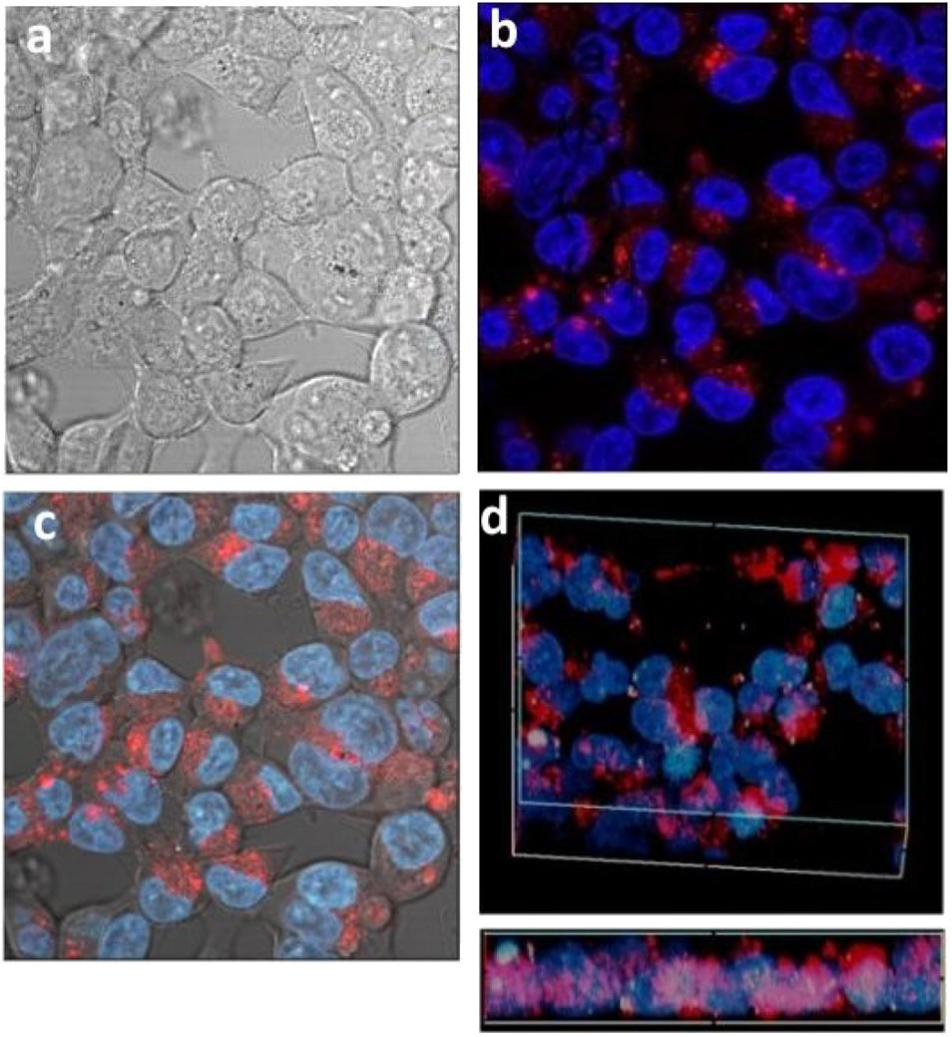
Confocal images of HEK293 cells incubated with small S-CμP-AA-Rh particles and co-stained with a DAPI dye. Bright field **(a)**, fluorescence confocal **(b)**, and combined **(c)**. Volume depth profile for particles incubated with transfection agent **(d)**.

Next the cellular uptake was investigated for the smaller particles S-CμP-AA-Rh by HEK293 cells. In contrast, some cellular internalization was observed for the smaller particles in the absence of the transfection agent (Figures 6a–c). However, the number of particles able to cross the cellular membrane appeared to be lower compared to the number when pre-incubated with the transfection agent (Figure 6d). Co-staining with DAPI, a nuclear stain, showed the particles to be in the cytoplasm and the perinuclear region. The strong localized emission observed indicates that while the transfection agent influences the number of particles internalized it does not influence the particle destination. Overall, in the presence of transfection agent no difference was observed between small and large particle in cellular uptake.

#### Cellular Delivery of a Model Payload by CμPs

**Figure 7:**
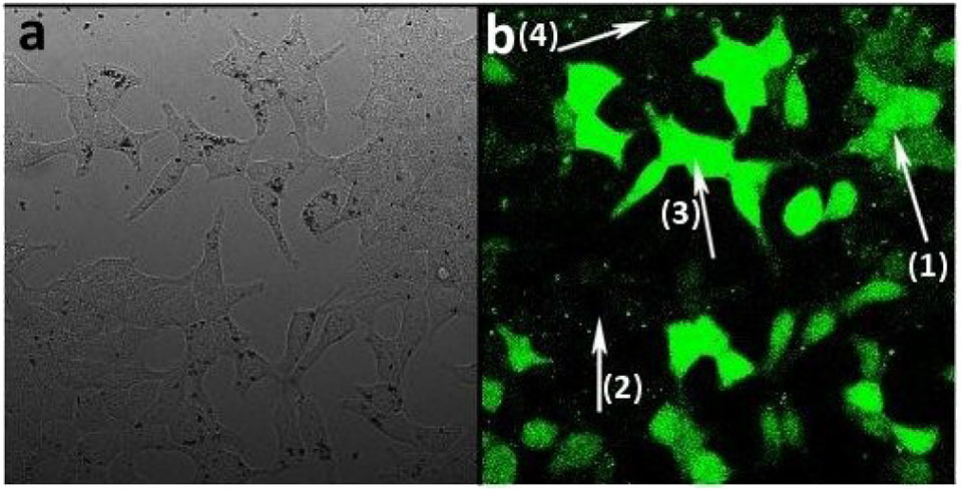
Confocal fluorescence microscopy images of HEK293 cells incubated with L-CμP-FL_ads_ particles **(a)** bright field image and **(b)** fluorescence image.

Activated carbons have been shown to be able to deliver drugs to cells (Valencia et al., 2011) and so having determined the conditions of cell uptake of porous microspheres we next investigated whether the prepared L-CμP-FL_ads_ particles were capable of delivering a model cargo into the cells (Kim et al., 2008). The bright field image recorded for the particles after incubation overnight with HEK293 cells show that particles are clearly visible in the cytoplasm of some cells while other cells show an absence of carbon particles (Figure 7a). These two populations are found to contrast dramatically when imaged in fluorescence mode (Figure 7b). In this image a significant number of cells display strong green emission throughout the cell cytoplasm and nucleus (**1**). This emission is attributed to the release of the dye molecules. In contrast, those cells that showed no evidence of particle presence in the bright field image remain “dark” (**2**). It is also notable that the most intensely fluorescent cells correspond to those cells that have a higher number of particles present in the bright field image (**3**). The presence of sharp, localized emission throughout the image is also noteworthy (**4**). This is attributed to CμP-FL_ads_ particles located outside the cell environment; in the case of these particles the dye molecule, due to its poor solubility in aqueous solution, remains adsorbed at the particle. The distribution of the fluorescein dye throughout the cell including the nucleus contrasts strongly the images recorded for L-CμP-FL particles that are covalently modified with fluorescein (Supplementary Figure 4). The widespread distribution is similar to that observed for the delivery of a fluorescein dye by SWNTs (Nakayama-Ratchford et al., 2007). The desorption of FL-NH_2_ is favored in the lipophilic intracellular medium; therefore, particle uptake and membrane transport is expected to trigger desorption of the dye within the cytoplasm. The most likely mechanism for particle uptake is endocytosis which has been observed for similarly sized particles (Pirollo and Chang, 2008). This mechanism of uptake will be the subject of future experiments.

While carbonaceous nanomaterials show promise for a range of biomedical applications, there are ongoing discussions about their toxicity (Firme, 2010; Zhao and Liu, 2012). *In vivo* and *in vitro* toxicological studies reveal differing toxicity among carbonaceous materials. For example, toxicity of single walled carbon nanotubes (SWNT) and multi walled carbon nanotubes (MWNTs) has been linked to their fibrous nature (Sharifi et al., 2012). In the case of MWNTs an increased risk of cancer has been linked to the long rigid structure (Poland et al., 2008) and the presence of metal impurities (Takagi et al., 2008). On the other hand, carbon nanohorns which share the sp^2^ covalent scaffold with nanotubes, but are free from metal impurities and possess a spherical shape (Iijima et al., 1999; Miyawaki et al., 2008), exhibit negligible toxicity compared to SWNT (Warheit et al., 2004) and MWNTs (Poland et al., 2008; Takagi et al., 2008; Sharifi et al., 2012; Zeinabad et al., 2016). This evidences the major role that aspect ratio (Wang et al., 2014) and impurities play in determining the toxicity of carbonaceous nanomaterials. In the case of our CμPs the combination of the metal free synthesis and spherical size can be considered significant factors in their biocompatibility. In fact, microscopic inspection of the cells incubated with a loading concentration of 2 μg of CμPs, showed an intact cell membrane over the course of the experiment (24 h). The cells were therefore found to tolerate the particles with no evidence of cell death in the images recorded for the different systems.

## Conclusions

CμPs are promising delivery agents with the potential to load large amounts of small molecules. Furthermore, their modification can be readily achieved using the wealth of carbon coupling chemistry. In this study the cellular uptake of large (690 nm) and small (250 nm) dye-labeled porous CμPs by HeLa and HEK293 cell lines was investigated by confocal fluorescence microscopy. In the presence of transfection agent both large and small microparticles were readily internalized by both cell lines. However, in absence of the transfection agent the uptake was influenced by both the particle size and the surface functionalization. In addition, we showed that in the case of the dye loaded particles cellular uptake is accompanied by dye release and delivery throughout the cell. We believe that this result paves the way for further exploitation of these versatile particles for applications in drug delivery. Furthermore, the recent discovery of incandescent behavior by porous carbon particles (Duffy et al., 2012) offers the potential for temperature-activated release of bioactive compounds or materials.

## Data Availability Statement

The raw data supporting the conclusions of this article will be made available by the authors, without undue reservation, to any qualified researcher.

## Author Contributions

PD prepared the particles. LM performed the particle modification and cell studies and DH performed particle characterization. RY prepared the cells used for the studies. AW and SB contributed to microscopy measurements. SQ and PC conceived the project and supervised the work. All authors contributed to the preparation of material for the paper and writing of the manuscript.

## Conflict of Interest

The authors declare that the research was conducted in the absence of any commercial or financial relationships that could be construed as a potential conflict of interest.
