# Supplementary material for: Porous Carbon Microparticles as Vehicles for the Intracellular Delivery of Molecules
Source: Front Chem. 2020 Oct 14;8:576175. doi: 10.3389/fchem.2020.576175 (PMC7591791; doi:10.3389/fchem.2020.576175)
Supplement: Supplementary file 1 [file Data_Sheet_1.PDF]

Supplementary Material

Porous Carbon Microparticles as Agents for Cell Delivery

L. M. Magno,[1] D. T. Hinds,[1] P. Duffy,[2] R. B. Yadav,[3] A. D. Ward,[3] S. W. Botchway,[4] P. E. Colavita[2] and S. J. Quinn*[1]

[a]School of Chemistry, University College Dublin, Dublin 4, Ireland.

[b]School of Chemistry, Trinity College Dublin, Dublin 2, Ireland.

[c]*Central Laser Facility, Science & Technology Facilities Council, Rutherford Appleton Laboratory, Didcot, Oxfordshire, OX11.*

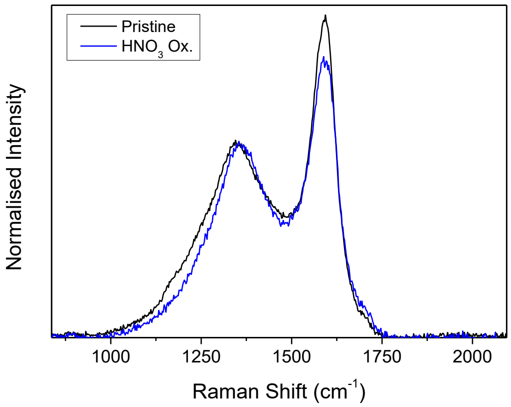

Supplementary Figure 1. Normalised, background subtracted spectra Raman spectra of pristine (black trace) and nitric acid oxidised (blue trace) L-CμP-COOH. Spectra recorded with 514 nm excitation with a 50 × objective lens.

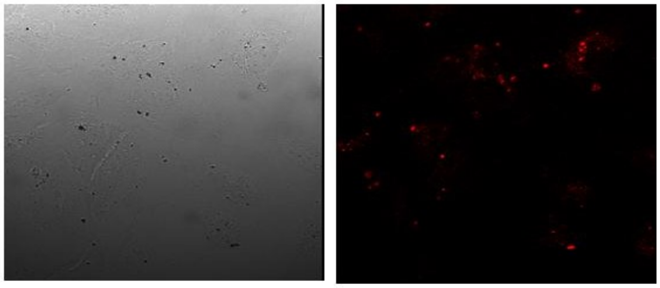

Supplementary Figure 2. Confocal images of HeLa cells incubated with large L-CμP-AAD-Rh particles in the absence of transfection agent (left) bright field image and (right) fluorescence image.

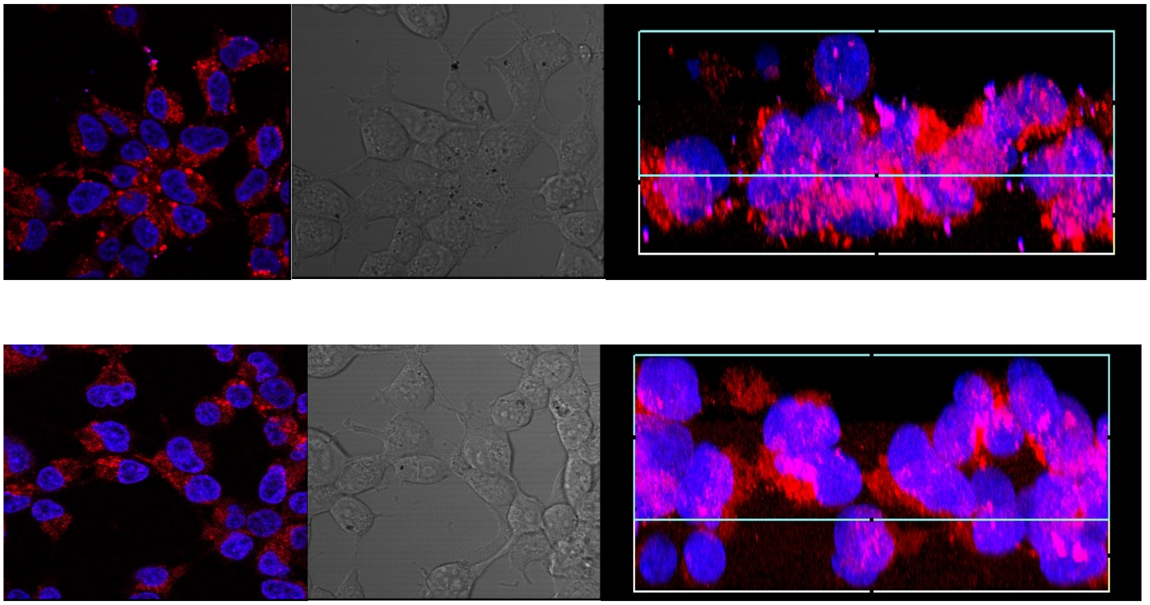

Supplementary Figure 3. Confocal images of HEK293 cells incubated with large L-CμP-PEG-Rh particles in the presence of transfection agent (top) and without transfection agent (bottom). (left) fluorescence image and (right) bright field image with associated depth profile analysis.

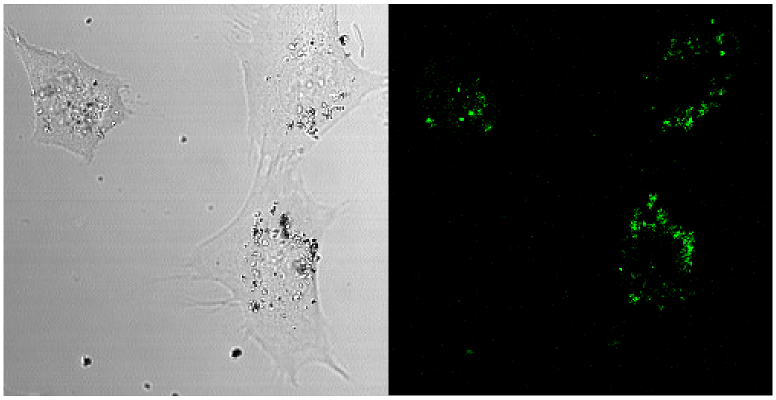

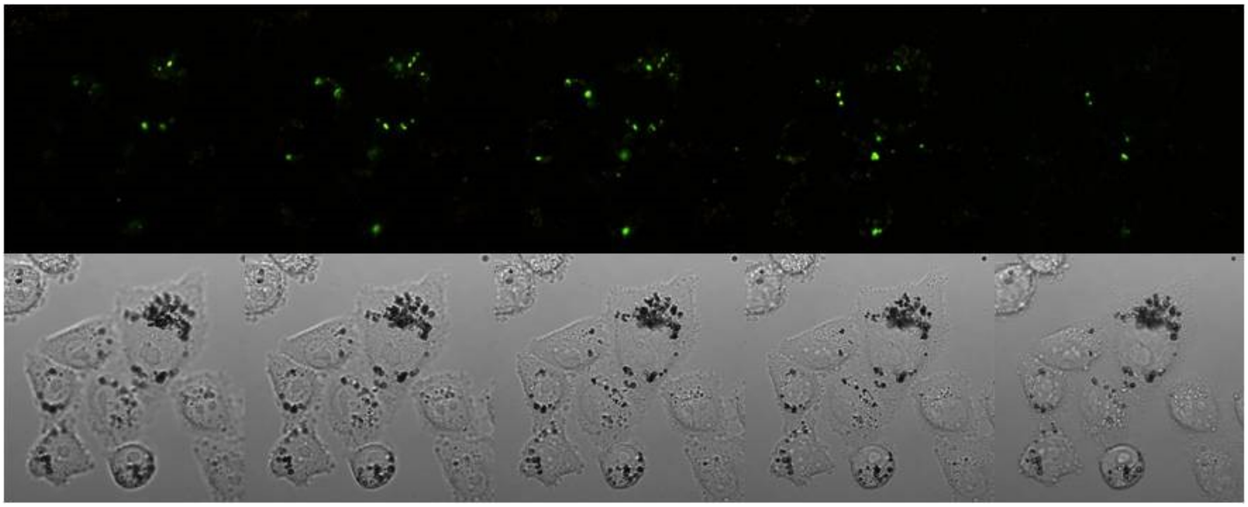

Supplementary Figure 4. Confocal images of HEK293 cells incubated with L-CμP-FL particles bright field image (left) and fluorescence image (right). Lower Confocal images of HeK293 cells after incubation with L-CμP–FL. (top) Sample scans showing changes in particle fluorescence with z-depth. (bottom) Bright field image showing L-CμP–FL in the cells.
